# Gender differences in symptom presentation and treatment outcome in children and youths with eating disorders

**DOI:** 10.1186/s40337-021-00468-8

**Published:** 2021-09-15

**Authors:** Jennifer S. Coelho, Janet Suen, Sheila Marshall, Alex Burns, Josie Geller, Pei-Yoong Lam

**Affiliations:** 1 Provincial Specialized Eating Disorders Program for Children and Adolescents, BC Children’s Hospital, 4500 Oak St., Vancouver, BC V6H 3N1 Canada; 2 Department of Psychiatry, University of British Columbia, 2255 Wesbrook Mall, Vancouver, BC V6T 2A1 Canada; 3 School of Social Work, University of British Columbia, 2080 West Mall, Vancouver, BC V6T 1Z2 Canada; 4 Division of Adolescent Health and Medicine, Department of Pediatrics, University of British Columbia, Vancouver, BC Canada; 5 Eating Disorders Program, St. Paul’s Hospital, Vancouver, BC Canada

**Keywords:** Pediatric, Eating disorders, Gender, Treatment outcomes, Muscularity concerns, Eating pathology

## Abstract

**Background:**

To address the gaps in the literature examining eating disorders among males and gender minority youths, a prospective study was designed to assess gender differences in eating disorder symptom presentation and outcomes. Muscularity concerns may be particularly relevant for male youths with eating disorders, and were included in assessment of eating disorder symptom presentation.

**Methods:**

All cisgender male youths who presented for specialized eating disorder treatment at one of two sites were invited to participate, along with a group of matched cisgender females, and all youths who did not identify with the sex assigned to them at birth. Youths completed measures of eating disorder symptoms, including muscularity concerns, and other psychiatric symptoms at baseline and end of treatment.

**Results:**

A total of 27 males, 28 females and 6 trans youths took part in the study. At baseline, Kruskal–Wallis tests demonstrated that trans youths reported higher scores than cisgender male and female youths on measures of eating pathology (Eating disorder examination-questionnaire (EDE-Q) and the body fat subscale of the male body attitudes scale (MBAS)). These analyses demonstrated that there were no differences between cisgender male and female youths on eating disorder symptoms at baseline. However, repeated measures ANOVA demonstrated that males had greater decreases in eating pathology at discharge than did females, based on self-reported scores on the EDE-Q, MBAS, and Body Change Inventory.

**Conclusions:**

Gender differences in eating pathology appeared at baseline, with trans youths reporting higher levels of eating pathology than cisgender youths, though no differences between cisgender males and females emerged at baseline for eating disorder symptom presentation. Contrary to expectations, there were no gender differences in measures of muscularity concerns. Males demonstrated greater eating disorder symptom improvements than females, suggesting that male adolescents may have better treatment outcomes than females in some domains.

## Plain English summary

Research in the field of eating disorders has focused on females, and less is known about the symptom presentation and treatment outcomes in males and gender minority youths. This prospective study set out to assess gender differences in eating pathology and treatment outcomes. Muscularity concerns may be particularly relevant for male youths with eating disorders, yet there is limited research on gender differences in muscularity concerns in treatment-seeking youths with eating disorders. Participants include cisgender male youths, matched cisgender females, and gender diverse youths who did not identify with the sex assigned to them at birth. Youths completed measures of eating disorder symptoms, including muscularity concerns, and other psychiatric symptoms at baseline and end of treatment. Some gender differences in eating pathology appeared at baseline, with trans youths reporting higher levels of eating pathology than cisgender youths. There were no differences between cisgender males and females in eating disorder symptom presentation at baseline, and contrary to expectations, there were no gender differences in measures of muscularity concerns. However, males demonstrated greater eating disorder symptom improvements than females.

## Overview and background

The predominance of research on females with eating disorders has led to gaps in knowledge about eating disorder symptom presentation in males and gender minorities. Murray and colleagues [33] highlight the marginalization of males from eating disorder research, and suggest that the gaps in research contribute to disparities in the assessment and diagnosis of males and gender minorities. The focus on females in the field of eating disorders has also been reported in research on eating disorder prevention, in which both males and gender minorities tend be excluded from research [14]. In the development of guidelines for virtual care in the context of the COVID-19 pandemic, Couturier and colleagues [15] proposed to examine how sex and gender impact virtual care outcomes for children, adolescents, and emerging adults with eating disorders. However, they noted very small numbers of male participants in the studies reviewed, which precluded the ability to provide evidence-based recommendations for sex and gender.

Within the limited literature on male eating disorders, there appears to be a higher proportion of pediatric males seeking eating disorders services in comparison to adult males. Adult eating disorders services report that males represent as little as 5% of referrals [6], with a review suggesting a range of adult male patients of between 5 and 11% [48] of those presenting to treatment. In contrast, there appears to be a larger proportion of males represented in early onset eating disorders in pediatric settings. There was a higher proportion of males with eating disorders who were under age 13, with males representing 16.5% of those who presented to a specialized eating disorder service, in comparison to 7.8% of adolescents aged 13–19 years [38]. Similarly, 25% of an Australian sample of children with early onset eating disorders were boys [29]. The reason for the higher proportion of young males in pediatric services relative to adult services is not clear, however, a population-based study in Poland suggests that eating disorders occur primarily in young men between the ages of 11–30 years [25]. A recent study on prevalence of eating disorders in adolescents indicated that the point prevalence of eating disorders in adolescent boys was 12.8% [30], supporting the need to ensure representation of males in eating disorder research and dispelling the myth that eating disorders in males are rare.

There are some demographic and clinical differences between male and female children and adolescents who present to eating disorder services. Male youths are typically younger than females at admission, are more likely to identify with an ethnic minority group, and have a higher likelihood of having an “atypical” or “other” eating disorder diagnosis [10, 26, 44]. However, some of these differences are not reported universally, with other pediatric samples of youths with eating disorders reporting no age differences between males and females (e.g., [53]). Studies report conflicting evidence with respect to the prevalence of co-occurring mental health conditions in male and female youths with eating disorders. Some studies report that male adolescents with eating disorders are more likely than females to have depression [42], whereas other studies indicated that female adolescents had higher rates of mood disorders [26]. A study on matched male and female adults with eating disorders reported that males had lower levels of obsessive–compulsive and depressive symptoms than did females [47]. However, a recent review highlighted conflicting evidence on comorbidity in males and females with eating disorders, indicating that some studies show higher rates of comorbidity in males but that this is not consistent across studies [28].

There are also differences in the eating disorder symptoms reported by males compared to females. Male adolescents with eating disorders report lower scores on the Eating Disorder Examination in comparison to females [26, 44]. A review focused on adolescents and young adults suggests males report more concerns about muscularity, and less shape and weight concern, drive for thinness, and body dissatisfaction than females [28]. Sex-related differences in clinical presentation need to be interpreted with caution, however. In a critical review of the literature, Murray and colleagues [33] highlight the marginalization of males in eating disorder research and emphasize the female-centric nature of eating disorder classification. Some males may be more likely to idealize a muscular body type, as opposed to the thin ideal, which may in turn drive differences in eating disorder symptom presentation across gender [36]. Furthermore, female young adults commonly report goals of weight loss, whereas a higher proportion of males report a goal to maintain their weight [9]. Chu and colleagues [9] highlight that there may be gender differences in weight control behaviors, with males potentially focusing on developing muscularity, and females focusing on leanness.

Muscularity-related concerns are not captured in the most common validated questionnaires in eating disorders, which tend to focus on thinness, and body parts that may be more relevant to females (e.g., hips and buttocks; [33]). Muscularity concerns and muscle-enhancing behaviors are higher in male adolescents and young adults than females [5, 35]. Muscularity concerns may also drive compulsive exercise behaviors. Male adults with anorexia nervosa reported higher levels of exercise pathology than did females with anorexia nervosa [32], though the measure used in this study (the Compulsive Exercise Test, [49]) focused on leanness-oriented exercise as opposed to muscularity-oriented exercise. In a sample of youths with eating disorders who completed both qualitative interviews and validated measures of eating disorder symptoms, boys highlighted the dialectic between leanness and muscularity, and were more likely than girls to attribute the development of eating disorder symptoms to sports and performance [3]. However, the small sample of boys in this study did not score higher than females on measures of obligatory exercise or drive for muscularity. Given the limited research on muscularity concerns and drive for exercise in pediatric populations with eating disorders, it is not yet clear whether there are gender differences in treatment outcomes for these symptoms.

The research on sex and gender differences in treatment outcome for eating disorders generally suggests that males and females respond similarly to treatment. A recent secondary analysis of randomized controlled trials investigated gender differences in treatment outcome, which demonstrated that boys with anorexia nervosa respond similarly to girls [22]. In a large study of males treated in a male-only treatment group, good outcomes were reported in both adolescents and adults [54]. Part of the challenge in interpreting single studies on outcomes in males is the small samples sizes across many studies. A systematic review in which data from a total of 1129 males with anorexia nervosa (including adults) were examined indicated that no solid conclusions can yet be made regarding treatment outcomes given the limited research and the frequent exclusion of males from analyses [46]. However, some studies suggest better treatment outcomes in males than females with eating disorders (e.g., [4, 45, 47]).

Many of the papers examining differences between males and females do not indicate whether they were assessing sex assigned to participants at birth, or gender, and whether there were any participants who did not identify with the sex assigned to them at birth. Gorrell and colleagues [22] indicated that they were not able to examine whether participants in their study identified with sex assigned to them at birth, as only gender was assessed. Therefore, it is difficult to distinguish whether existing research focuses on cisgender male youths. A recent scoping review demonstrated that youths who do not identify with the sex assigned at birth are at risk for the development of eating disorders [12]. In alignment with the definition used in the scoping review, trans individuals are defined as those whose gender is not aligned with the sex assigned to them at birth, including transgender, non-binary, and gender diverse individuals [27]. The scoping review highlighted that the literature on trans youths with eating disorders is limited to case studies and retrospective chart reviews, with few papers including assessment of eating disorder and related symptoms with validated measures [12]. Given the limited research on eating disorder symptoms in gender diverse populations, it is not clear whether standard measures such as the Eating disorder examination-questionnaire (EDE-Q; [18]) have adequate psychometric properties for use with trans youths. Recent research supports the use of global scores (but not subscale scores) on the EDE-Q to assess eating pathology in trans youths, and suggests that trans youths report similar levels of eating pathology as cisgender females [39]. Youth who identified with a gender other than male or female also demonstrated a prevalence of eating disorders similar to females, and a higher prevalence than males [30]. However, there are not yet studies on the efficacy of eating disorder treatments for gender minority populations, and a lack of evidence on treatments for sexual and gender minority individuals due to lack of appropriate measurement of gender, and inadequate inclusion in research studies [8].

To address the gaps in the literature examining eating disorders among males and gender minority youths, a prospective study was designed to assess gender differences in eating disorder symptom presentation and outcomes. The current study also examined symptoms of depression and obsessive–compulsive disorder, given research on adults reporting differences in both these symptoms among males and females [47]. Measures that assess male-relevant aspects of eating pathology, including muscularity concerns, were included in accordance with recommendations by Darcy and Lin [17]. We hypothesized that males would score higher than females on measures that included assessment of symptoms that may be more relevant to males, such as muscularity concerns, and that males would report lower scores on the EDE-Q, as well as on measures of depression and obsessive–compulsive symptoms. We expected all youths would report decreases in eating disorder symptoms over the course of treatment. Given the limited data on the eating disorder outcomes in gender minority youths, this study also represents an exploratory study to investigate eating disorder and related symptom presentation in trans youths.

## Methods

### Participants

All cisgender male youths who were admitted to the Provincial Specialized Eating Disorders Program for Children and Adolescents at the British Columbia (BC) Children’s Hospital or Looking Glass Residence between September 2015 and February 2019 were invited to participate. Male participants were matched with the next cisgender female who met matching criteria outlined by the research team and was admitted to the program.^1^ If a matched female declined the study invitation, an invitation was extended to the next admission who met the matching criteria. Matching criteria included: symptom presentation (restrictive vs. binge-eating/vomiting, and body image concerns present or absent), age (within 2.5 years at the time of admission), and treatment type (outpatient eating disorders at BC Children’s Hospital, intensive day or inpatient treatment at BC Children’s Hospital, or residential treatment at the Looking Glass Residence). All trans youths (including transgender, non-binary, pangender, and other gender diverse individuals) who were admitted to the programs were also invited to participate. Parents/caregivers of participants were also invited to take part. Given the lack of availability of validated translations of the study measures across languages, proficiency in English was required to participate. In the event that parents of youths were not proficient in English, but youths were, an interpreter was available to translate information from the consent form for parents/caregivers.

### Procedure

Potential participants (i.e., male youths, trans youths, and females who were matched to a male youth) who provided permission to be approached by the research team were provided with information about the study around the time of their admission to the program. Participants who were receiving treatment at the Looking Glass Residence who had reached the age of majority (19 years) provided signed consent, as did parents/caregivers. Children and adolescents ages 18 and under provided signed assent. Participants were asked to complete measures at baseline (at assessment/admission to the program), discharge,^2^ and 3-month follow-up. The current report focuses on baseline and discharge data. Medical records of participants were also accessed to determine details about eating disorder and co-occurring psychiatric diagnoses, anthropometric measurements, vital signs and bloodwork results, and details about clinical presentation at assessment. All participants had a diagnosis of an eating disorder in accordance with criteria from the fifth edition of the Diagnostic and Statistical Manual of Mental Disorders (DSM-5; [2].

BC Children’s Hospital Provincial Specialized Eating Disorders Program for Children and Adolescents typically offers treatment for those between 7 and 18 years, while Looking Glass Residence offers specialized eating disorders treatment for those between 16 and 24 years. An overview of the clinical programs offered in the Provincial Specialized Eating Disorders Program at BC Children’s Hospital and the Looking Glass Residence are reported by Coelho and colleagues [10].

Upon obtaining assent and consent, participants were provided with a link to complete study measures online via Research Electronic Data Capture (REDCap), which is hosted at the BC Children’s Hospital Research Institute. REDCap is a secure, web-based application designed to support data capture for research studies [24]. Families could either choose to complete the measures at home after receiving the link by e-mail, or complete these measures in the program with a tablet that was provided to them by the research team. Families could also receive a paper version of the measures if they preferred this option. Families who participated in the study were offered a gift card with a value of $10 at each timepoint for which they completed measures.

Data in the current report focus on data from the youths, parent data will be reported separately. Data from interviews with parent participants in the study have been previously reported [13], and body checking data from a portion of the sample were reported as part of a separate research question [11].

### Measures

Youths were asked to complete a demographics questionnaire, which included questions about the sex assigned to them at birth, gender identity, and ethnicity. Participants were also provided with questionnaires that assessed both eating disorder and general psychiatric symptoms.

#### Eating disorder symptom measures

*Eating disorder examination-questionnaire* (EDE-Q, adapted for adolescents, [31]^3^): This measure is an adaptation of the original EDE-Q [18], which has been modified with language appropriate for adolescents. This measure provides an assessment of eating pathology, and taps into concerns relating to dietary restraint, eating, shape and weight. A global score was calculated based on the average of the 22 subscale items [1], with a range of possible scores between 0 and 6 with higher scores indicating higher levels of eating pathology. The scale has adequate psychometric properties, including good internal consistency for both male and female adolescents [31], and a unifactorial solution based on the global score has been reported to be supported for trans youths [39]. Cronbach’s alpha for the sample in this study was 0.95 for females, 0.94 for males, and 0.95 for trans youths.

*Body change inventory* (BCI; [40]): This 18-item measure assesses strategies used by adolescents to change their body size/shape. This measure includes assessment of strategies to decrease body size, as well as increase body size and increase muscle size. Responses are rated on a 5-point scale, with total scores based on a sum of all items. The range of potential total scores is 18–90. The psychometric properties of the scale have been established with school-based samples of adolescent girls and boys [40]. Cronbach’s alpha for the total score in this study was 0.94 for females, 0.88 for males, and 0.72 for trans youths.

*Male body attitudes scale-revised* (MBAS; [43]): This 15-item measure was designed to assess body image concerns relevant to males, and addressed some of the psychometric concerns of the original version developed by Tylka et al. [51]. This scale has been validated in a community-based sample of male young adults, and has been demonstrated to have good psychometric properties. The measure assesses concerns about body fat (score range 5–25), muscularity (score range 7–35) and height (score range 3–15), with items for each of the subscales averaged, and a total score based on a sum of all items. Higher scores indicate greater levels of negative body attitudes. Although this measure is specific to male body image concerns, the language used is gender neutral, and the authors have suggested that this measure could be employed to assess differences between males and females [51]. Cronbach’s alpha for the total score in this study was 0.86 for females and 0.90 for males. A negative average covariance was obtained for the sample of trans youths, suggesting low reliability of the total score in this sample. Therefore, the three subscale scores were examined, which all demonstrated adequate reliability across the groups (Cronbach’s alpha ranged from 0.63 to 0.95).

*Obligatory exercise questionnaire* (OEQ; [50]): This is a 20-item measure used to access exercise frequency, and attitudes towards exercise. Responses on this measure are on a 4-point Likert scale, and are summed to provide a total score ranging from 20 to 80, with higher scores indicating higher levels of compulsive exercise. Cronbach’s alpha for the sample in this study was 0.95 for females, 0.92 for males, and 0.96 for trans youths.

#### Psychiatric symptom measures

*Centre for epidemiological studies-depression scale (revised)* (CES-D; [16]): This brief, well-validated measure provides an assessment of depressive symptoms. The revised scale has been demonstrated to have good validity with high internal reliability [52]. Total scores range from 0 to 60, with higher scores indicating more depressive symptoms. Cronbach’s alpha for the sample in this study was 0.92 for females, 0.92 for males, and 0.65 for trans youths.

*Obsessive–compulsive inventory-child version* (OCI-CV; [20]): This 21-item measure assesses obsessive–compulsive symptoms in children and youths. Total scores range from 0 to 42, with higher scores indicating higher levels of obsessive–compulsive symptoms. Cronbach’s alpha for the sample in this study was 0.90 for females, 0.90 for males, and 0.89 for trans youths.

### Statistical analyses

Scores on self-report questionnaires were calculated if less than 10% of items were missing, with averaging of available items employed to generate subscale and total scores if there was missing data. Non-parametric tests (Kruskal–Wallis) were conducted to assess differences between male, female, and trans youths at Time 1 for continuous measures, given unequal sample sizes across groups and violations of normality for some measures based on Kolmogorov–Smirnov test (EDE-Q for males and trans youths, OEQ for female youths, and MBAS subscales for trans youths). Chi-square analyses were employed to test group differences for categorical measures. The alpha level was set at *p* < 0.05. Descriptive statistics, including mean and standard deviation (for normally distributed variables), and median and interquartile range (for non-normally distributed variables) are also reported.

Treatment outcome data in male and female youths (changes from Time 1 to Time 2) were analyzed with repeated measures analysis of variance (ANOVA). A decision was made to conduct an available case analysis, given the small sample of male youths, and the fact that data were available for all participants for the majority of the primary measures for the 20 male and 20 female participants who completed both Time 1 and Time 2 measures (*n* = 19 males, 18 females had scores at both Time 1 and Time 2 for EDE-Q, no missing total scores for other 3 primary eating disorder measures, and for psychiatric symptoms, 19 males had data available for OCI-CV at both time points, data were available at both time points on the OCI-CV for all females, with no missing scores for the CES-D). There were violations of the assumption of normality for some of the measures at Time 2 (EDE-Q, BCI and OCI-CV for males, and EDE-Q and OEQ for females), but given that sample size was similar across groups, and values for skewness and kurtosis were less than 2 for all measures, repeated measures ANOVA was considered to be an acceptable approach. Given the small sample of trans youths, there was insufficient power to analyze changes in this group. Means and percentage change are presented for comparative purposes. None of the measures for trans youths had violations of normality at Time 2. Analyses were conducted using SPSS Statistics version 26.

## Results

### Participant characteristics

A total of 27 males, 28 females, and 6 trans youths participated in the study. Trans youths included those who identified as non-binary/genderqueer (*n* = 1), transgender female (*n* = 1), and transgender male (*n* = 4). There were an additional four males who were admitted to BC Children’s Hospital or the Looking Glass Residence during the study period who declined to participate, representing a 87% participation rate for males. Consent and assent were obtained for one male participant who subsequently disengaged from treatment and did not complete questionnaires. Five females declined to participate (85% participation rate), and one female participant completed only one measure at Time 1. No trans youths declined to participate. Eating disorder diagnoses and other demographic and clinical information for the sample are presented in Table 1. Matching for males and females was based on symptom presentation (presence or absence of body image concerns, and binge eating/vomiting), therefore there are some differences in the diagnoses across males and females. There were no significant group differences across male, female and trans youths in those who reported body image concerns (Fisher’s exact test reported due to low expected count in 33% of the cells, *p* = 0.79). Differences in age across male, female and trans youths did not reach statistical significance, *H*(2) = 4.86, *p* = 0.088. No significant group differences emerged for ethnicity [Fisher’s exact, *p* = 0.49],^4^ nor were there group differences in duration of illness at the time of assessment (*H*(2) = 0.63, *p* = 0.73).

**Table 1:** Demographic and clinical features of participants at time 1

	Males (n = 27)	Females (n = 28)	Trans (n = 6)
Treatment location	BC Children’s Hospital: 26	BC Children’s Hospital: 27	BC Children’s Hospital: 5
	Looking glass: 1	Looking glass: 1	Looking glass: 1
Eating disorder diagnosis at admission	AN-R: 14	AN-R: 14	AN-R: 2
	AN-BP: 2	BN: 2	AN-BP: 1
	Unspecified eating disorder: 2	OSFED (Atypical BN): 1	BN: 2
	ARFID: 9*	Unspecified eating disorder: 3	ARFID: 1
		ARFID: 8	
Body image concerns	Yes: 17	Yes: 18	Yes: 5
	No: 10	No: 10	No: 1
Age	*M* = 14.23 years, *SD* = 2.58 (9–18 years)	*M* = 14.30 years, *SD* = 2.42 (9–20 years)	*M* = 16.88 years, *SD* = 2.62 (14–21 years)
Ethnicity	Caucasian: 15 (55.6%)	Caucasian: 15 (53.6%)	Caucasian: 5 (83.3%)
	Ethnic minority: 12 (44.4%)	Ethnic minority: 13 (46.4%)	Ethnic minority: 1 (16.7%)
Duration of illness (months)	Median = 9.39, IQR = 27.33 (2.10–159.85)	Median = 7.46, IQR = 11.66 (1.81–72.17)	Median = 11.47, IQR = 45.27 (2.89–94.61)
Symptom measures^a^			
Eating disorder examination-questionnaire (EDE-Q)	2.41, IQR = 3.00 (0.09–4.81)	2.48, IQR = 3.39 (0.27–5.54)	4.91, IQR = 1.33 (2.14–5.55)
Male body attitudes scale (MBAS)			
Height	6.50, IQR = 5.00 (3.00–13.00)	7.00, IQR = 5.00 (3.00–12.00)	14.00, IQR = 7.75 (5.00–15.00)
Muscularity	15.00, IQR = 11.75 (7.00–35.00)	16.00, IQR = 9.00 (7.00–29.00)	17.50, IQR = 15.25 (16.00–35.00)
Body fat	12.50, IQR = 12.80 (5.00–25.00)	13.00, IQR = 11.00 (5.00–25.00)	24.50, IQR = 2.80 (17.00–25.00)
Obligatory exercise questionnaire (OEQ)	45.50, IQR = 27.25 (23.00–71.00)	41.00, IQR = 33.00 (20.00–75.00)	54.00, IQR = 34.75 (27.00–70.00)
Body change inventory (BCI)	40.50, IQR = 22.00 (18.00–75.00)	50.00, IQR = 31.00 (18.00–84.00)	55.50, IQR = 14.00 (42.00–68.00)
Centre for epidemiological studies-depression scale (CES-D)	20.50, IQR = 24.84 (1.00–54.00)	33.00, IQR = 23.00 (0.00–57.00)	43.50, IQR = 14.00 (38.00–52.00)
Obsessive–compulsive inventory-child version (OCI-CV)	6.00, IQR = 10.00 (0.00–22.00)	16.00, IQR = 16.37 (4.00–30.00)	22.00, IQR = 17.50 (9.00–34.00)

Descriptive statistics, including mean and standard deviation (for normally distributed variables), and median and interquartile range (for data that was not normally distributed) are reported. Median and interquartile range are presented for symptom measures given non-normal distribution of scores on some measures. The range of scores/values is presented in brackets

^*^One youth had a diagnosis of ARFID when first admitted to the program, but this diagnosis changed to Anorexia nervosa—restrictive subtype early in the admission, and matching was performed based on diagnosis at the time of matching

^a^There were missing data for some measures at Time 1. A total of 26 females completed the EDE-Q, and 27 females completed the remaining measures (MBAS, OEQ, BCI, CES-D, and OCI-CV). A total of 25 males completed the EDE-Q, and OCI-CV, and 26 completed the remaining measures (OEQ, BCI, and CES-D). There were no missing data for trans youth for measures at Time 1

### Eating disorder symptomatology

Group differences emerged for EDE-Q global scores, *H*(2) = 9.12, *p* = 0.010. Pairwise comparisons demonstrated that trans youths reported higher scores than did male youths (*p* = 0.003) or female youths (*p* = 0.037). No differences emerged between male and female youths (*p* = 0.149). Scores on symptom measures at Time 1 are presented in Table 1.

Scores on the MBAS subscales were examined separately, given problems with the internal consistency of total scores on this measure. Bonferroni correction (0.05/3) was applied for the subscale analysis, to control for alpha inflation given that the a priori analytic plan was to examine the total score on this measure. Group differences emerged on the body fat subscale score, *H*(2) = 9.71, *p* = 0.008. Pairwise comparisons demonstrated that trans youths reported higher scores than did either male (*p* = 0.002) or female (*p* = 0.008) youths. No differences emerged between male and female youths on the body fat subscale. Group differences on the muscle subscale (*H*(2) = 2.69, *p* = 0.26) and height subscale (*H*(2) = 6.36, *p* = 0.042) did not reach Bonferroni-corrected significance.

No group differences emerged on scores on either the BCI, *H*(2) = 3.59, *p* = 0.167, or the OEQ, *H*(2) = 1.03, *p* = 0.598.

Given the relatively large proportion of participants who did not report body image concerns, follow-up analyses were performed in which only those who endorsed body image concerns were included. The pattern of results did not change with this subanalysis.^5^

### Psychiatric symptom measures

Group differences emerged on the OCI-CV, *H*(2) = 13.50, *p* = 0.001. Pairwise comparisons demonstrated that male youths reported lower scores than did either female (*p* = 0.002) or trans (*p* = 0.004) youths. No differences emerged between female and trans youths (*p* = 0.31). Significant group differences also emerged on the CES-D, *H*(2) = 13.75, *p* = 0.001. Trans youths reported higher scores than did females (*p* = 0.04), who in turn reported higher scores than males (*p* = 0.02). The difference between males and trans youths also reached significance (*p* = 0.001).

### Evaluation of treatment outcome data

A chi-square analysis was performed to examine if there were any predictors of attrition at Time 2. All trans youths (n = 6) completed Time 2 measures, and were not part of the repeated measures analyses so were not included in analyses of predictors of attrition. There were no differences in the proportion of males (n = 20, 74.1% of initial sample) and females n = 20, 71.4% of initial sample) who completed measures at Time 2 [*χ*^2^ (1, N = 55) = 0.05, *p* = 0.83, Phi = 0.03], nor were there significant differences in the proportion of youths who reported body image concerns who took part at Time 2 [*χ*^2^ (1, N = 55) = 2.57, *p* = 0.11, Phi = 0.22]. No differences in length of treatment for males and females emerged for the sample of 40 participants who completed measures at Time 1 and Time 2, U = 249.50, *p* = 0.18. Details of the cisgender male and female participants who completed both Time 1 and Time 2 are provided in Table 2.

**Table 2:** Demographic features of participants who completed Time 1 and Time 2

	Males (n = 20)	Females (n = 20)
Treatment location	BC Children’s Hospital: 19	BC Children’s Hospital: 19
	Looking glass: 1	Looking glass: 1
Eating disorder diagnosis at admission	AN-R: 12	AN-R: 12
	AN-BP: 2	BN: 1
	Unspecified eating disorder: 1	Unspecified eating disorder: 3
	ARFID: 5	ARFID: 4
Body image concerns	Yes: 14	Yes: 14
	No: 6	No: 6
Age	*M* = 14.35 years (*SD* = 2.80)	*M* = 14.54 years (*SD* = 2.29)
Ethnicity	Caucasian: 12 (60.0%)	Caucasian: 11 (55.0%)
	Ethnic minority: 8 (40.0%)	Ethnic minority: 9 (45.0%)
Length of treatment	Median: 149.0 days	Median: 100.5 days
	IQR: 268.0	IQR: 130.0

There was a significant effect of time on participants’ percentage median Body Mass Index (mBMI) from Time 1 to Time 2, *F*(1,38) = 121.85, *p* < 0.001, partial η^2^ = 0.76, with the mean mBMI of the sample increasing from 86.80% (SD = 12.09) to 99.35% (SD = 11.32). There was not a significant interaction with gender, *F*(1,38) = 1.28, *p* = 0.26, partial η^2^ = 0.03.

### Treatment outcome: eating disorder symptoms

Mean scores across treatment for males, females, and trans youths are reported in Table 3. Due to a violation of equality of covariance and equality of error variance, data on the EDE-Q were log transformed. Repeated measures analyses demonstrated a significant effect of time on EDE-Q scores, *F*(1,35) = 20.97, *p* < 0.001, partial η^2^ = 0.38, as well as a significant interaction between time and gender, *F*(1,35) = 4.24, *p* = 0.047, partial η^2^ = 0.11. Paired t-tests on raw EDE-Q scores were conducted to further examine the interaction, which demonstrated that males had a significant decrease in EDE-Q scores from Time 1 to Time 2 (*t*(18) = 4.59, *p* < 0.001, *d* = 1.15), whereas females did not demonstrate a significant change in scores (*t*(17) = 1.39, *p* = 0.18, *d* = 0.34). A similar pattern of results was obtained for MBAS total scores, F(1,38) = 5.23, *p* = 0.028, partial η^2^ = 0.12, as well as a significant interaction between time and gender, F(1,38) = 4.24, *p* = 0.046, partial η^2^ = 0.10. Paired t-tests were conducted to examine the interaction, which demonstrated that males had a significant decrease in MBAS scores from Time 1 to Time 2 (*t*(19) = 2.92, *p* = 0.009, *d* = 0.61), whereas females did not demonstrate a significant change in scores (*t*(19) = 0.17, *p* = 0.86, *d* = 0.03).

**Table 3:** Mean scores (with standard deviations) on eating disorder and psychiatric symptom measures at Time 1 and Time 2

	Males (n = 20)	Females (n = 20)	Trans (n = 6)
Eating disorder examination-questionnaire (EDE-Q)^a^			
Time 1	2.34 (1.59)	2.99 (1.83)	4.52 (1.22)
Time 2	0.72 (0.91)	2.14 (1.94)	3.62 (1.61)
Male body attitudes scale (MBAS)			Not reported (given concerns with internal consistency)
Time 1	36.80 (13.71)	39.30 (12.11)	
Time 2	29.19 (10.10)	38.90 (15.16)	
Obligatory exercise questionnaire (OEQ)			
Time 1	44.97 (15.52)	46.92 (17.52)	51.50 (17.42)
Time 2	33.90 (7.57)	42.74 (17.83)	45.67 (19.57)
Body change inventory (BCI)			
Time 1	41.85 (12.46)	46.50 (20.51)	55.17 (9.26)
Time 2	28.62 (10.77)	39.80 (16.49)	46.33 (22.64)
Centre for epidemiological studies-depression scale (CES-D)			
Time 1	20.51 (14.10)	29.15 (13.28)	44.50 (6.50)
Time 2	10.40 (8.26)	23.15 (15.99)	39.50 (16.10)
Obsessive–compulsive inventory-child version (OCI-CV)^a^			
Time 1	9.00 (7.09)	15.24 (8.48)	20.33 (9.67)
Time 2	6.53 (7.48)	14.65 (12.24)	17.50 (7.45)

^a^There were missing data on the EDE-Q and OCI-CV for some participants. A total of 19 males and 18 females completed the EDE-Q at both Time 1 and Time 2, and 19 males completed the OCI-CV at both time points

Due to a violation of equality of error variance, and a marginally significant violation of equality of covariance (*p* = 0.054) total scores on the BCI were log transformed. Repeated measures analyses demonstrated a significant effect of time on BCI scores, *F*(1,38) = 20.16, *p* < 0.001, partial η^2^ = 0.35, as well as a significant interaction between time and gender, *F*(1,38) = 4.82, *p* = 0.034, partial η^2^ = 0.11. Paired t-tests on raw BCI scores were conducted to examine the interaction, which demonstrated that males had a significant decrease in scores from Time 1 to Time 2 (*t*(19) = 5.51, *p* < 0.001, *d* = 1.13), whereas females did not demonstrate a significant change, *t*(19) = 1.71 *p* = 0.10, *d* = 0.35.

Due to a violation of equality of covariance and equality of error variance, total scores on the OEQ were transformed. Log transformation corrected the violation of equality of covariance (but not the equality of error variance). Repeated measures analyses demonstrated that there was a significant effect of time on OEQ scores, *F*(1,38) = 11.61, *p* = 0.002, partial η^2^ = 0.23, with scores decreasing from Time 1 to Time 2, but no significant interaction between time and gender, *F*(1,38) = 1.98, *p* = 0.17, partial η^2^ = 0.05.

Trans youths reported decreases of 20% on the EDE-Q global scores from Time 1 to Time 2, 11% on the OEQ, and 16% on the BCI. Cisgender female youths reported similar reductions on the EDE-Q (28%), OEQ (9%), and BCI (14%). The percentage reductions for cisgender males were 69% on the EDE-Q, 25% on the OEQ, and 32% on the BCI.

### Treatment outcome: psychiatric symptoms

Due to a violation of equality of covariance and equality of error variance, total scores on the OCI-CV and CES-D were log transformed. Repeated measures analyses demonstrated that there was a significant effect of time on OCI-CV scores, *F*(1,37) = 10.35, *p* = 0.003, partial η^2^ = 0.22, but no significant interaction between time and gender, *F*(1,37) = 1.06, *p* = 0.31, partial η^2^ = 0.03. Similarly, there was a significant effect of time on CES-D scores, *F*(1,38) = 9.91, *p* = 0.003, partial η^2^ = 0.21, but no significant interaction between time and gender, *F*(1,38) = 2.14, *p* = 0.15, partial η^2^ = 0.05.

Trans youths reported decreases of 11% on the CES-D and 14% on the OCI-CV from Time 1 to Time 2. In contrast, cisgender female youths reported reductions of 21% on the CES-D and 4% on the OCI-CV, while cisgender male youths reported 49% reduction on the CES-D and 27% reduction on the OCI-CV.

## Discussion

Trans youths reported higher levels of eating pathology on the EDE-Q, as well as the body fat subscale of the MBAS at Time 1, relative to cisgender males and females. Contrary to expectations, no differences between males and females emerged on the MBAS or the BCI, which assess male-relevant concerns including muscularity and strategies to increase body size/muscle size. No group differences emerged on the OEQ, a measure of obligatory exercise. In line with hypotheses, males reported lower levels of depression and obsessive–compulsive symptoms than did females, and also lower scores than trans youths. The lower levels of psychiatric symptoms reported by males in the current study aligns with previous reports that adult males have lower depressive and obsessive–compulsive symptoms [47]. The lack of gender differences on the muscularity subscale of the MBAS, or scores on the measure of obligatory exercise mirror findings from a small study of matched male and female adolescents with eating disorders [3].

As expected, there were significant decreases in both eating disorder and psychiatric symptoms over the course of treatment. There were no differences in treatment outcome between males and females on the psychiatric measures of depressive and obsessive–compulsive symptoms, nor were there differences on measures of obligatory exercise. Similarly, both males and females demonstrated an increase in percentage body mass index over the course of treatment. However, males demonstrated a better outcome than females on some of the eating disorder symptom measures, including the EDE-Q, MBAS, and BCI. The better outcomes in males than females aligns with some previous reports (e.g., [4, 45, 47]). It is noteworthy that males, but not females, demonstrated decreased scores on the MBAS and BCI, given that these measures have been proposed to assess concerns that particularly relevant to males. This finding indicates that muscularity concerns, and other concerns that have been thought to be specific to males are also relevant to females and may in fact be less amenable to change in females. Murray and colleagues [33] have highlighted the importance of not conflating muscularity concerns with male eating disorders, and called for additional research on muscularity concerns in females.

Given the inclusion of children and young adolescents in the current study, some differences in muscularity concerns may have been obscured. A large study of male adolescents and young adults demonstrated that muscularity concerns emerge in mid to late adolescence [7]. Similarly, muscle-enhancing behaviors and exercise for muscle enhancement peaks in early adulthood (ages 20–22) for males [35]. As a result, it is possible that muscularity concerns may be higher in samples of older adolescents and young adults with eating disorders. A total of 30% of the females and 35% of males who completed Time 2 data in the current study were aged 13 or under. Clinical and gender differences have previously been reported in children with eating disorders in comparison to adolescents [38]. Furthermore, the measures chosen to assess muscularity-related concerns (the MBAS and BCI) have not yet been validated in adolescents with eating disorders. The BCI was chosen as it was among the measures highlighted by Darcy and Lin [17] that had good psychometric properties in adolescents. Similarly, the MBAS was chosen due to its proposed measures of male-specific body image concerns that included muscularity, as well as height and body fat [43]. However, newly published measures such as the muscularity-oriented eating test [34] may better capture how pursuit of muscularity impacts eating behavior.

This study is one of the first to report on treatment-related changes in measures of eating disorder and other psychiatric symptoms in a sample of trans youths. It is noteworthy that trans youths reported significantly higher eating disorder symptoms than did cisgender males or females at Time 1 as measured by the EDE-Q. Trans youths who participated in large surveys in Youth Health or College Assessment surveys in public schools and colleges also reported higher levels of eating disorder symptoms relative to cisgender youths (see [12] for review). Although the small sample size of trans youths precluded analysis of treatment-outcomes, a reduction in eating disorder and psychiatric symptoms was noted from Time 1 to Time 2 when looking at aggregate scores on measures. The reduction in eating disorder symptomatology was similar in trans youths and cisgender females. Recent research has supported the potential utility of the EDE-Q for trans youths [39], when using the global score as calculated in the current study. However, concerns about the internal validity of the MBAS arose, given a lack of internal consistency for the group of trans youths who participated in the study. Further research is needed to validate measures of eating disorder symptoms with gender minority individuals.

No group differences in duration of illness emerged in the current study. Some studies have highlighted that males with eating disorders report delayed recognition of the eating disorder and difficulties accessing treatment [41]. Males are also more likely to have an undiagnosed eating disorder, which has been attributed to perceived stigma and reluctance to seek treatment [23]. Although the median duration of illness did not differ between male, female, and trans participants in the current study, there was a large range, with some individuals having eating disorder symptoms for years prior to accessing tertiary level eating disorder services. Conflicting findings across the lifespan have emerged, as some studies with adults report an older age of onset and older age at first presentation to treatment in males compared to females (e.g., [19]), whereas pediatric studies generally report males are younger than females at admission and/or have an earlier age of onset [10, 26, 44]. In line with the current findings, these pediatric studies also report no differences between males and females in duration of illness [10, 26, 44]. However, the treatment setting differs across studies, as some research is based on inpatients (e.g., [19, 47], some studies based on outpatients (e.g., [26]), and some studies based in services offering a continuum of care (e.g., [10, 44]). There appear to be limited studies in secondary level, community-based eating disorder treatment settings. It is possible that presenting features across gender may differ across different service types.

### Strengths and limitations

The current study matched male and female participants according to key clinical criteria, including symptom presentation, body image concerns, and age. The decision was made to match on symptom presentation, rather than diagnosis, given some of the challenges in differentiating between anorexia nervosa and avoidant restrictive food intake disorder, and the diagnostic shift that is reported in a portion of adolescents with eating disorders [37]. The sample size of cisgender males who completed both Time 1 and Time 2 data is similar to the number of males who participated in two large randomized clinical trials [22], and this paper is the first to report symptom measures and treatment outcome in a sample of trans youths in comparison to cisgender youths with eating disorders. The response rate of cisgender male and trans individuals was high, suggesting that the current sample was representative of those who seek treatment in the two sites for this study. There was also diversity in the ethnicity of participants, with over 40% of cisgender males and females representing an ethnic minority group.

Limitations of the study include the small sample size of male, female, and trans youths. Attrition at Time 2 was between 26 and 28% for cisgender males and females, and there were missing data on some measures for youths at both time points. Longer term follow-up (i.e., 12–24 months after treatment) is needed to assess whether gender differences persist over time. There were also challenges in matching some males to females, including delays in finding females who met matching criteria, meaning that youths did not always overlap in the timing of their treatment. The matching also did not account for changes in treatment plan and intensity or the course of treatment (e.g., some youths stepped up to a higher level of care during treatment, and therefore did not necessarily receive the same treatment as their match). The timing of participants’ completion of Time 1 and Time 2 measures also did not always align with the dates of their admission and discharge, with some participants requiring several reminders to complete the measures. Furthermore, the small sample of trans youths, and differing sample sizes across cisgender and trans youths, led to non-parametric tests being conducted to assess gender differences at Time 1 and precluded the inclusion of trans youths in repeated measures analyses.

## Conclusions and future directions

The current study demonstrates that there are limited differences between cisgender males and females in eating disorder symptom presentation at baseline. However, males demonstrated greater eating disorder symptom improvements than females, in alignment with some previous reports. Longer-term follow-up is needed to establish whether these improvements persist over time. Trans youths demonstrated higher scores on measures of eating pathology relative to cisgender youths. There was a lack of gender differences in measures of muscularity concerns, and a lack of treatment-related changes on measures that assessed muscularity and other male-relevant concerns in cisgender females. Future research elucidating the nature of muscularity concerns in cisgender females with eating disorders, and how these concerns may differ from those of males, is warranted. Additional research establishing the psychometric properties of eating disorder symptom measures in trans youths is also warranted.

## Data Availability

The data are available from the corresponding author on reasonable request.

## Abbreviations

ANOVA: Analysis of variance
AN-R: Anorexia nervosa - restrictive subtype
AN-BP: Anorexia nervosa - binge-eating/purging subtype
ARFID: Avoidant restrictive food intake disorder
BC: British Columbia
BCI: Body change inventory
BN: Bulimia nervosa
CES-D: Centre for epidemiological studies-depression scale (revised)
DSM-5: Diagnostic and statistical manual of mental disorders, fifth edition
EDE-Q: Eating disorder examination-questionnaire
mBMI: Median body mass index
MBAS: Male body attitudes scale-revised
OCI-CV: Obsessive–compulsive inventory-child version
OSFED: Other specified feeding or eating disorder
OEQ: Obligatory exercise questionnaire
REDCap: Research electronic data capture

## Footnotes

1 One participant was recruited as a male and matched to a female, but disclosed identifying as female after admission. The matched female was retained in analyses, leading to one additional female compared to the male group. There was also a female who was initially matched to a male who was in the consent process, but who ultimately did not participate in the study. This female was re-matched to another male who was subsequently admitted to the program. Finally, there were two sets of twins (one set of male twins and one set of female twins) who were recruited into the study, who were matched to each other, and who were treated as independent participants for the purposes of data analysis.

2 Discharge was defined as the completion of primary eating disorder treatment goals. There were some cases who continued attending services in the program after primary goals were completed, primarily due to lack of available community services at the time of planned discharge. One youth had on-going, infrequent contact with staff in the program, with no plans for discharge—a decision was made to collect data for the second timepoint after 2.5 years in the program for anyone who was not yet discharged.

3 The first five participants completed a different version of this measure, the Youth EDE-Q [21]. Although there are some minor wording differences across the measures, the 22 questions for the subscale items overlap. Responses on the Youth EDE-Q version were included in analyses.

4 Information about ethnicity was extracted from clinical records, as the demographic form that included self-reported ethnicity was implemented part way through the study. One participant did not have information about ethnicity available in the chart, but had completed the self-reported measure so the self-reported information was included for this participant.

5 The group differences on global scores of the EDE-Q remained significant. The significant results established for the body fat subscale score on the MBAS fell below the Bonferroni-corrected threshold of 0.016 after excluding those without body image concerns (*H*(2) = 7.39, *p* = 0.025), but the direction of effects was the same. All other results held the same pattern when excluding those without body image concerns.
